# Knowledge and self-reported practices of infection control among various occupational groups in a rural and an urban hospital in Vietnam

**DOI:** 10.1038/s41598-018-23462-8

**Published:** 2018-03-23

**Authors:** La Thi Quynh Lien, Nguyen Thi Kim Chuc, Nguyen Quynh Hoa, Pham Thi Lan, Nguyen Thi Minh Thoa, Emilia Riggi, Ashok J. Tamhankar, Cecilia Stålsby Lundborg

**Affiliations:** 1 Health Systems and Policy (HSP): Improving the Use of Medicines, Department of Public Health Sciences, Karolinska Institutet, Tomtebodavägen 18A, 17177 Stockholm, Sweden; 2 Department of Pharmaceutical Management and Pharmaco-economics, Hanoi University of Pharmacy, 13-15 Le Thanh Tong, Hoan Kiem district, 110403 Hanoi Vietnam; 3 Department of Family Medicine, Department of Dermatology and Venereology, Hanoi Medical University, 01 Ton That Tung, Dong Da District, 116516 Hanoi Vietnam; 4 National Centralized Drug Procurement Centre – Vietnam Ministry of Health, 138A Giang Vo street, Ba Dinh district, 118401 Hanoi Vietnam; 5 Department of Brain and Behavioral Sciences, Unit of Medical Statics and Genomics, University of Pavia, 27100 Pavia, Italy; 6 Indian Initiative for Management of Antibiotic Resistance, Department of Environmental Medicine, R.D. Gardi Medical College, Agar Road, 456006 Ujjain, India

## Abstract

Staff practice, driven by knowledge, plays a decisive role in hospital infection control. This study aimed to assess and compare knowledge and self-reported practices of infection control among various occupational groups in a rural and an urban hospital in Vietnam. Questionnaires consisting of items on knowledge and practices were collected from 339 hospital staff with varying occupations. For analysis, total knowledge or practice score ranged from 0–15. Mood’s median test was performed to compare median scores. Post-hoc analysis of ordinal logistic regression models was applied to test differences in scores among occupational groups. The majority of hospital staff had good or adequate knowledge (median score: rural = 11.8; urban = 12), but the score range was wide (1.4–14.5). Self-reported practices in the urban hospital were likely to be better than in the rural one (*p* = 0.003). Self-reported practices yet not completely satisfactory, indicating the need for continuing professional development in both settings. Overall, cleaners had lower scores than both physicians and nurses, highlighting the need for tailored education in this topic. Future infection control strategies within the hospitals might want to assess the difference between the staff’s self-reported practice and their actual real practice. These findings can be of value in many other similar settings.

## Introduction

Inadequate infection control favours the spread of microorganisms in healthcare facilities, that might cause healthcare-associated infections (HAIs)^1^. HAIs aggravate the patient’s general health status, resulting in additional prescription of antibiotics, leading to increased costs for patients and the healthcare system, as well as antibiotic resistance^2^. In Europe, it is estimated that HAIs contribute to 37,000 excess deaths and approximately €7 bn additional direct costs each year. Data are scarce from low- and middle-income countries (LMICs), where the situation is likely worse with more devastating impacts^3^.

In an individual healthcare facility, staff practice, which is driven by their knowledge and attitudes, plays a decisive role in the success of infection control programmes^4^. A review concluded that “compliance to infection control precautions is internationally suboptimal”^5^. The core problem is not the lack of effective precautions and evidence-informed guidelines, but that healthcare workers (HCWs) apply these measures inadequately and inconsistently^5,6^. Particularly in LMICs, the problem of inadequate performance of HCWs is urgent^7^. An important determinant of the high burden of HAIs in LMICs is paucity of knowledge and lack of application of basic infection control measures^3,8^. Identifying existing knowledge and practices of infection control among HCWs is the first step in developing successful infection control programmes.

This study aimed to assess and compare staff knowledge and self-reported practices of infection control among varying qualification groups in a rural and an urban hospital in Vietnam where high prevalence of HAIs in hospitals has been reported^9,10^.

## Results

### Demographic characteristics

The study participants’ demographic characteristics are described in Table 1. Mean age was 35.8 ± 11.1 years, with females being the dominant sex (rural hospital: 81.8%, urban hospital: 89.9%), and nurses/midwives being the dominant qualification group (rural hospital: 66.2%, urban hospital: 67.4%) followed by physicians (rural hospital: 31.0%, urban hospital: 24.9%) and cleaners (rural hospital: 2.8%, urban hospital: 7.8%).

**Table 1:** Study participant’s demographic characteristics.

Characteristics	Total (N = 339) n (%)	Rural hospital (n = 144) n (%)	Urban hospital (n = 195) n (%)
**Age (35.8 ± 11.1)**	**(n = 292)**	**(n = 129)**	**(n = 163)**
20–30	144 (49.3)	78 (60.5)	66 (40.5)
31–40	52 (17.8)	18 (13.9)	34 (20.9)
41–50	46 (15.8)	13 (10.1)	33 (20.2)
51–60	50 (17.1)	20 (15.5)	30 (18.4)
**Sex**	**(n = 326)**	**(n = 137)**	**(n = 189)**
Male	44 (13.5)	25 (18.2)	19 (10.1)
Female	282 (86.5)	112 (81.8)	170 (89.9)
**Qualification**	**(n = 335)**	**(n = 142)**	**(n = 193)**
Physicians	92 (27.4)	44 (31.0)	48 (24.9)
Nurses/midwives	224 (66.9)	94 (66.2)	130 (67.4)
Cleaning workers	19 (5.7)	4 (2.8)	15 (7.8)

### Staff’s knowledge and self-reported infection control practices

Staff’s knowledge and self-reported infection control practices are presented in Table 2.

**Table 2:** Staff’s knowledge and self-reported infection control practices.

Assessment	Rural hospital (n = 144)	Urban hospital (n = 195)	*p*-value
**Knowledge score (median (range))**	**11.8 (6.8–13.9)** **n (%)**	**12 (1.4–14.5)** **n (%)**	**0.173**
Poor knowledge	1 (0.7)	3 (1.5)	
Adequate knowledge	49 (34.0)	49 (25.1)	
Good knowledge	94 (65.3)	143 (73.4)	
**Practice score (median (range))**	**11.4 (4.7–15.0)** **n (%)**	**12.4 (1.0–15.0)** **n (%)**	0.003*
Poor practice	3 (2.1)	2 (1.0)	
Adequate practice	68 (47.2)	35 (18.0)	
Good practice	73 (50.7)	158 (81.0)	

NOTE. *P*-values were extracted from Mood’s median test; *Significant at *p-*value < 0.05.

#### Knowledge

The median knowledge scores were 11.8 (6.8–13.9) and 12 (1.4–14.5) in the rural and urban hospitals respectively. The difference in median knowledge scores between the two hospitals was not statistically significant (*p* = 0.17). In both the hospitals, the majority of respondents showed good knowledge (rural hospital: 65.3%, urban hospital: 73.4%).

#### Self-reported infection control practices

The median practice scores were 11.4 (4.7–15.0) and 12.4 (1.0–15.0) in the rural and urban hospitals respectively. The difference in median of practice scores between the two hospitals was statistically significant (p = 0.003). Similar to the knowledge score, most staff scored good to adequate practice scores range in both hospitals.

### Comparison of knowledge and self-reported practices across occupational groups

#### Within the two hospitals

Comparisons of knowledge and practice scores across qualification groups within the studied hospitals are presented in Table 3. In both hospitals, cleaners had lower knowledge and practice score compared to physicians and nurses/midwives. The differences were statistically significant in the case of the urban hospital: Knowledge score: OR (95% CI): 0.13 (0.04–0.51), *p* = 0.001 and 0.12 (0.03–0.41), *p* < 0.001 compared physicians and nurses/midwives respectively; Practice score: OR (95% CI): 0.19 (0.06–0.67), *p* = 0.005 and 0.15 (0.05–0.46), *p* < 0.001 compared physicians and nurses/midwives respectively.

**Table 3:** Comparison of knowledge and practice scores across qualification groups.

Comparison	Knowledge score			Practice score		
	OR	95% CI	*p*-value	OR	95% CI	*p*-value
**Rural hospital**						
Nurses/midwives vs. Physicians	0.76	0.31–1.82	0.99	1.33	0.52–3.37	0.99
Cleaning workers vs. Physicians	0.14	0.02–1.22	0.09	0.48	0.06–4.00	0.99
Cleaning workers vs. Nurses/midwives	0.18	0.02–1.55	0.17	0.37	0.05–2.82	0.71
**Urban hospital**						
Nurses/midwives vs. Physicians	1.14	0.52–2.51	0.99	1.30	0.59–2.88	0.99
Cleaning workers vs. Physicians	0.13	0.04–0.51	0.001*	0.19	0.06–0.67	0.005*
Cleaning workers vs. Nurses/midwives	0.12	0.03–0.41	<0.001*	0.15	0.05–0.46	<0.001*

NOTE. *P*-values were adjusted using Bonferroni correction; *Significant at *p*-value < 0.05.

#### Between the two hospitals

The comparisons of knowledge and practice scores within qualification groups between the two studied hospitals are presented in Table 4. Nurses/midwives had 2.52 higher odds of having better knowledge scores in the urban hospital than in the rural hospital (OR (95% CI): 2.52 (1.49–4.26), *p* < 0.001), and 3.4 times higher for the practice scores (OR (95% CI): 3.4 (2.01–5.85), *p* < 0.001). Physicians working in the urban hospital had 3.5 times higher odds of achieving higher practice scores compared to physicians working in the rural hospital (OR (95% CI): 3.5 (1.55–7.93), *p* = 0.003).

**Table 4:** Comparison of knowledge and practice scores within qualification groups between the studied hospitals.

Comparison (Urban hospital vs. Rural hospital)	Knowledge score			Practice score		
	OR	95% CI	*p*-value	OR	95% CI	*p*-value
Physicians	1.67	0.77–3.57	0.19	3.5	1.55–7.93	0.003*
Nurses/midwives	2.52	1.49–4.26	<0.001*	3.4	2.01–5.85	<0.001*
Cleaning workers	1.60	0.24–10.57	0.62	1.4	0.23–8.43	0.71

**NOTE**. *Significant at *p*-value < 0.05.

### Self-reported reasons for non-compliance with hand hygiene

**Figure 1:**
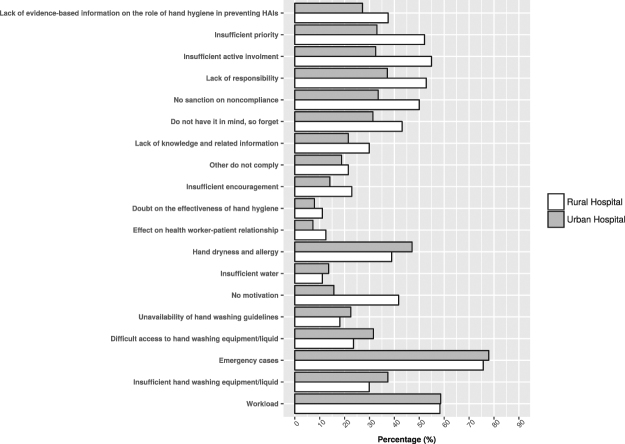
Self-reported reasons for non-compliance with hand hygiene.

Various reasons for non-compliance with hand hygiene were reported (Fig. 1). In both hospitals, the two leading reasons were emergencies (rural hospital: 75.7%, urban hospital: 75.9%) and high workload (rural hospital: 58.3%, urban hospital: 57.4%). Lack of equipment or soap was one of the most frequent reported reasons in the urban hospital, followed by dry hands and allergies.

## Discussion

To the best of our knowledge, no studies on staff knowledge and reported practices of hospital infection control in Vietnam have been published so far. No previous studies on the topic have been conducted with hospital cleaners alone nor combined with doctors and nurses. Additionally, most studies on infection control have been conducted in tertiary urban or teaching hospitals, with very few studies involving rural settings^11^. Our study has attempted to assess and compare staff knowledge and self-reported practice of hospital infection control targeting different occupational groups (physicians, nurses/midwives and cleaners) in both rural and urban settings.

A main result of our study was good knowledge of infection control among the majority of hospital staff. This was in contrast to the findings of similar studies from other low and middle-income countries (LMICs), such as Mongolia, Uganda, Nepal and Iran, where knowledge deficit has been recognised as one of the main barriers for the effective implementation of infection control programmes^12–15^. This can be explained by policies implemented by the Vietnamese Ministry of Health (MoH) to popularise and update documents for continued training in hospital infection control^16–18^. Nonetheless, some staff still showed poor knowledge and the knowledge score was wide, indicating the need for continuing professional development in both settings.

Among the staff, cleaners had lower knowledge scores than physicians and nurses/midwives. Non-significant results in the rural hospital can be due to low participation rates of cleaning workers. The role of cleaning staff in hospital infection control is usually underestimated although they themselves and their work can be a vector of infection transmission in hospitals. In fact, cleaning itself can be an important intervention in controlling HAIs in hospitals^19^. Therefore, there is a need for tailored education on this topic for cleaning staff in particular.

Reported practices in the urban hospital were likely to be better than in the rural one. This might be due to the fact that the rural hospital had poorer conditions for infection controls than the urban one. Although practice scores were good for the majority of respondents in both hospitals, the staff reported various reasons for non-compliance with hand hygiene, a simple but effective measure for infection prevention and control. This suggests that there could be a difference between the staff’s self-reported and actual practice.

In both hospitals, the staff considered emergencies and high workload the main reasons for their non-compliance. In emergencies, patients require quick examination. However, such patients can present with more severe health statuses making them more vulnerable to HAIs if hospital staff do not follow standard operating procedures for hygiene. High workload is often due to high patient overload. Making alcohol-based hand rub more readily available, for example at the patient’s bedside or in the staffs’ pockets, would make its use more feasible whenever needed.

Although good knowledge is a pre-requisite for a successful infection control program, it does not necessarily guarantee good practice. The know-do gap in infection control practice has been reported previously in various studies from LMICs for example Ethiopia and Nigeria^20–22^. It has been highlighted in a recent Vietnamese MoH scientific workshop on the topic that infection control in Vietnamese hospitals is of low-quality and does not properly comply with MoH regulations^23^. In many Vietnamese hospitals, effective infection control is difficult to achieve as most hospitals are old, overcrowded and have a high workload^24^. The possible difference between the staff’s self-reported practice and their actual practice might need to be considered in future infection control strategies.

This study’s strength lies in the fact that it was conducted with hospital staff of varying qualifications in both rural and urban settings, providing a more diverse perspective into the topic. However, since participants completed the questionnaires on their own without supervision, a number of questionnaires lacked good quality information and were therefore omitted from the final analysis. In addition, for analysis, the questions were assumed to be equally important which might not be true, needing further assessment. Overall, we believe that the findings of the study can be of value in many other similar settings.

## Methods

This cross-sectional study was conducted in a rural hospital and an urban hospital in Hanoi, Vietnam in 2013. The 220-bedded rural hospital employed 46 doctors, 110 nurses, 12 midwives and 12 cleaning staff, whilst the 520-beded urban hospital had 181 doctors, 392 nurses, 32 midwives and 35 cleaning staff.

### Data collection

A questionnaire consisting of a section on knowledge and another on practice, was used for data collection. It was adapted for the Vietnamese context based on the World Health Organization’s Practical Guide on Prevention of Hospital-acquired Infections 2^nd^ Edition (Malta, 2002), the Vietnam Ministry of Health’s Training Document on Infection Prevention and Control (Vietnamese, 2012), and the authors’ experience of conducting research in hospital infection control as well as using questionnaires^18,25,26^. Similar questionnaire was used by the research team in another setting^26^. The questionnaire was piloted with hospital staff for face validity. The questionnaire was piloted with 20 respondents following which few modifications were made. Data from the pilot study were not included in the final analysis. In addition to the participants’ demographic characteristics (age, sex, workplace, qualification), 15 items each for knowledge and practice were included in the questionnaire (Supplementary Info). A question on justification for non-compliance with hand hygiene, the most important preventive measure for infection control, was also included. The questions had closed response alternatives where the participant could select one or more alternatives as instructed.

Questionnaires were distributed to hospital staff ensuring to recruit staff with different qualifications (physicians, nurses, midwives, cleaners) from various departments. The questionnaires were then collected and assessed for data quality. Finally, 144 questionnaires from the rural hospital and 195 from the urban hospital were included in the analysis.

### Data analysis

#### Scoring of knowledge and practice

Each question was given a score from 0 to 1. For questions where only one alternative was possible, 1 point was given to a correct answer and 0 to an incorrect response. For questions where multiple alternatives could be chosen, 1 point was given if all alternatives were correct and 1/n points (n = the number of alternatives) for each alternative with a correct response. Knowledge or practice scores for each individual were calculated and summed up to attain the total score. The total knowledge or practice scores ranged from 0 to 15. Scores were divided by quartile. The first cut-off corresponded to the 2^nd^ quartile (7.5) and the second cut-off to the 3^rd^ quartile (11.25). Thus, a total score of <7.5 was considered poor knowledge/practice, 7.5 to <11.25 was considered adequate knowledge/practice, and ≥11.25 was considered good knowledge/practice.

#### Statistical analysis

Descriptive statistics were used to present participants’ demographic characteristics. Numerical variables were expressed as medians (including the range) while categorical variables were measured as percentages. Mood’s median test was performed to compare median scores. A post-hoc analysis of ordinal logistic regression models was applied to test the difference in scores among qualification groups within/between the two hospitals, adjusting for age and sex. Bonferroni correction was used to adjust *p*-values for multiple comparisons. All analyses were performed in R 3.3.1 using packages “RVAideMemoire”, “ordinal” and “lsmeans”^27^.

### Ethical approval

The study was approved by Hanoi Medical University Review Board in Bio-Medical Research (N0. 116/HMU IRB, 21^st^ December 2012). The methods were performed in accordance with relevant guidelines and regulations. Participants were informed about the study and that confidentiality will be maintained throughout. It was assumed that by answering the questionnaire, respondents consented to participate.

### Data availability

The datasets generated during and/or analysed during the current study are available from the corresponding author on reasonable request.

## Electronic supplementary material

Questionnaire
