# Supplementary material for: Knowledge and self-reported practices of infection control among various occupational groups in a rural and an urban hospital in Vietnam
Source: Sci Rep. 2018 Mar 23;8:5119. doi: 10.1038/s41598-018-23462-8 (PMC5865156; doi:10.1038/s41598-018-23462-8)
Supplement: Supplementary file 1 — Questionnaire [file 41598_2018_23462_MOESM1_ESM.docx]

# Knowledge and self-reported practices of infection control among various occupational groups in a rural and an urban hospital in Vietnam

La Thi Quynh Lien^1,2*^, Nguyen Thi Kim Chuc^3^, Nguyen Quynh Hoa^4^, Pham Thi Lan^3^, Nguyen Thi Minh Thoa^3^, Emilia Riggi^1,5^, Ashok J. Tamhankar^1,6^, Cecilia Stålsby Lundborg^1^

*^1^ Health Systems and Policy (HSP): Improving the Use of Medicines, Department of Public Health Sciences, Karolinska Institutet, Tomtebodavägen 18A, 17177 Stockholm, Sweden; Cecilia.Stalsby.Lundborg@ki.se*

*^2^ Department of Pharmaceutical Management and Pharmaco-economics, Hanoi University of Pharmacy, 13-15 Le Thanh Tong, Hoan Kiem district, 110403 Hanoi, Vietnam; lienltq@hup.edu.vn*

*^3^ Department of Family Medicine, Department of Dermatology and Venereology, Hanoi Medical University, 01 Ton That Tung, Dong Da District, 116516 Hanoi, Vietnam; chuc.ntk@gmail.com;* [*landhy2003@yahoo.com*](mailto:landhy2003@yahoo.com)*; ntmthoa2017@gmail.com*

*^4^ National Centralized Drug Procurement Centre – Vietnam Ministry of Health, 138A Giang Vo street, Ba Dinh district, 118401 Hanoi, Vietnam; quynhoa29@gmail.com*

*^5^ Department of Brain and Behavioral Sciences, Unit of Medical Statics and Genomics,* [*University of Pavia*](https://www.researchgate.net/institution/University_of_Pavia)*, Italy; emilia.riggi@gmail.com*

*^6^ Indian Initiative for Management of Antibiotic Resistance, Department of Environmental Medicine, R.D. Gardi Medical College, Agar Road, 456006 Ujjain, India;* [*ejetee@gmail.com*](mailto:ejetee@gmail.com)

*****Corresponding author: La Thi Quynh Lien, [lienltq@hup.edu.vn](mailto:lienltq@hup.edu.vn); Tel.: +46 (0) 704 363 771

**Questionnaire**

| **Knowledge items** |
| --- |
| 1. **What do you understand by HAIs?** |
| Patients have symptoms of infection upon admission |
| Infections occurring within 24 hours after admission |
| Infections occurring more than 48 hours after admission |
| Re-admission due to surgical site infections within 1 month after severe operations |
| Surgical site infections developing within 10 days after surgery |
| 1. **From the items listed below, what are symptoms of HAIs?** |
| Fever upon admission |
| Urinary tract infection due to catheterisation |
| Drainage from a surgical wound |
| Pneumonia upon admission |
| Fever after surgery |
| Diarrhoea 4 days after admission |
| Drainage at an injection site 5 days after admission |
| Umbilical infections 5 days after admission |
| Infections after ophthalmic surgery |
| Upper respiratory tract infections 4 days after admission |
| 1. **Which of the following are transmission pathways for HAIs?** |
| Via hospital staff |
| Via hospital environment (air, water, food, etc.) |
| Via medical equipment |
| Via patients |
| Via visitors |
| 1. **Is infection prevention and control important? (Yes/No/Do not know)** |
| 1. **What are the possible consequences of HAIs?** |
| Increased morbidity and mortality rates |
| Increased treatment costs |
| Increased financial burden for hospitals and patients |
| Increased use of antibiotics |
| Prolonged treatment course |
| 1. **Can practising infection control reduce the rate of HAI? (Yes/No/Do not know)** |
| 1. **What are the possible measures for infection prevention and control?** |
| Hand washing |
| Extensive antibiotic use |
| Use of gloves |
| Use of prophylactic vaccines |
| Antiseptic conditions in operating rooms |
| Maintaining hygiene of the hospital environment |
| 1. **What do you understand by cross-infections?** |
| Infections transferred from patient to patient |
| Infections upon admission |
| Infections transferred from hospital staff to patients |
| Infections transferred from patients to hospital staff |
| Infections transferred within hospitals |
| 1. **Isolation rooms are necessary for?** |
| Patients with contagious diseases |
| Patients with acquired multidrug-resistant bacteria |
| Patients with acquired Methicillin-resistant *Staphylococcus aureus* (MRSA) |
| HIV patients |
| Hepatitis B patients |
| Patients with measles, rubella or mumps |
| 1. **What are possible measures for prevention of HAIs related to catheterisation in hospitals?** |
| Treatment using antibiotics |
| Aseptic technique at insertion |
| Avoiding catheterisation when unnecessary |
| Limiting the duration of catheterisation |
| Longer duration of catheterisation |
| 1. **Which types of waste are so-called ‘healthcare waste’?** |
| Waste from diagnosis and treatment processes |
| Waste from sub-clinical testing |
| Waste from research activities |
| Waste causing toxic effects or disease spread from hospitals |
| Waste from the administration areas of hospitals |
| 1. **What are the risks of improper hospital waste management?** |
| Increased incidence of HAIs |
| Environmental pollution |
| Increased health risks for the healthcare workers |
| Violation of hospital practice management |
| Creating a bad image for the hospital |
| 1. **Choose the correct answers for proper waste classification.** |
| Red bags/boxes are for sharp objects such as injection needles and surgical knives |
| Blue or white bags/boxes are for water cans, juice cans, packages |
| Yellow bags/boxes are for gauzes/cloths which are blood-stained or contain bodily fluids |
| Black bags/boxes are for tubes, conductor lines, plastic catheters |
| Green bags/boxes are for placentas, body tissues and organs |
| 1. **When do you think is the proper time to classify healthcare waste? (choose only one)** |
| After all the waste in the ward is collected |
| Immediately at the waste bin |
| 1. **When do you think is the proper time to collect waste bags? (choose only one)** |
| When the bags are full |
| When the bags are half full |
| When the bags are ¾ full |
| **NOTE.** HAI: Healthcare-associated infection |

| **Practice items** |
| --- |
| 1. **Are data on HAIs routinely collected at your hospital? (Yes/No/Do not know)** |
| 1. **Did you ever receive information on HAIs in your hospital? (Yes/No/Do not know)** |
| 1. **Does your hospital have a department of infection control? (Yes/No/Do not know)** |
| 1. **How many times do you wash your hands at work per day? (Choose only one)** |
| Less than five times  Five times or more |
| 1. **At any one time, for how long do you wash your hands?**   Less than one minute  About one minute or more |
| 1. **With what do you wash your hands?** |
| Water only |
| Water and soap/hand washing liquid/alcohol hand rub |
| 1. **Why do you wash your hands?** |
| To prevent myself from getting an infection |
| To prevent myself and my patients from getting an infection |
| 1. **Is waste classified at your ward? (Yes/No/Do not know)** |
| 1. **If yes, when is the waste classified at your ward?** |
| After all the waste in the ward has been collected |
| At each waste bin |
| 1. **What bags do you have at your ward for waste classification?** |
| Red |
| Blue/white |
| Yellow |
| Black |
| Green |
| Not classified |
| No bags/bins for waste |
| 1. **What bags do you use for plastic waste such as catheters and infusion lines?** |
| Red |
| Blue/White |
| Yellow |
| Black |
| Green |
| Not classified |
| No bags/bins for waste |
| 1. **In which bags do you put expired medicines?** |
| Black |
| Yellow |
| Blue/white |
| Green |
| Any bag |
| 1. **When are waste bags are collected at your ward?** |
| When the bags are full |
| When the bags are half full |
| When the bags are ¾ full |
| 1. **How often are waste bags collected at your ward?** |
| Within 24 hours |
| Once per day to once per week |
| Longer than once week |
| 1. **In the last 3 years did you participate in any training on hospital infection control? (Yes/No/Do not know)** |
| **NOTE.** HAI: Healthcare-associated infection |
